# Communication through Resonance in Spiking Neuronal Networks

**DOI:** 10.1371/journal.pcbi.1003811

**Published:** 2014-08-28

**Authors:** Gerald Hahn, Alejandro F. Bujan, Yves Frégnac, Ad Aertsen, Arvind Kumar

**Affiliations:** 1 Unité de Neuroscience, Information et Complexité (UNIC), CNRS, Gif-sur-Yvette, France; 2 Faculty of Biology, and Bernstein Center Freiburg, University of Freiburg, Freiburg, Germany; The University of Chicago, United States of America

## Abstract

The cortex processes stimuli through a distributed network of specialized brain areas. This processing requires mechanisms that can route neuronal activity across weakly connected cortical regions. Routing models proposed thus far are either limited to propagation of spiking activity across strongly connected networks or require distinct mechanisms that create local oscillations and establish their coherence between distant cortical areas. Here, we propose a novel mechanism which explains how synchronous spiking activity propagates across weakly connected brain areas supported by oscillations. In our model, oscillatory activity unleashes network resonance that amplifies feeble synchronous signals and promotes their propagation along weak connections (“communication through resonance”). The emergence of coherent oscillations is a natural consequence of synchronous activity propagation and therefore the assumption of different mechanisms that create oscillations and provide coherence is not necessary. Moreover, the phase-locking of oscillations is a side effect of communication rather than its requirement. Finally, we show how the state of ongoing activity could affect the communication through resonance and propose that modulations of the ongoing activity state could influence information processing in distributed cortical networks.

## Author Summary

The cortex is a highly modular structure with a large number of functionally specialized areas that communicate with each other through long-range cortical connections. It is has been suggested that communication between spiking neuronal networks (SNNs) requires synchronization of spiking activity which is either provided by the flow of neuronal activity across divergent/convergent connections, as suggested by computational models of SNNs, or by local oscillations in the gamma frequency band (30–100 Hz). However, such communication requires unphysiologically dense/strong connectivity, and the mechanisms required to synchronize separated local oscillators remain poorly understood. Here, we present a novel mechanism that alleviates these shortcomings and enables the propagation synchrony across weakly connected SNNs by locally amplifying feeble synchronization through resonance that naturally occurs in oscillating networks of excitatory and inhibitory neurons. We show that oscillatory stimuli at the network resonance frequencies generate a slowly propagating oscillation that is synchronized across the distributed networks. Moreover, communication with such oscillations depends on the dynamical state of the background activity in the SNN. Our results suggest that the emergence of synchronized oscillations can be viewed as a consequence of spiking activity propagation in weakly connected networks that is supported by resonance and modulated by the dynamics of the ongoing activity.

## Introduction

The brain processes sensory stimuli by an organized flow of neuronal activity across a distributed network of specialized cortical areas. This flow requires mechanisms that route neuronal signals from one cortical area to another. However, the exact nature of this routing process remains poorly understood.

Experimental studies suggest that synchronization of spiking activity may play a pivotal role in the flow of neuronal activity, as synchronous neuronal firing can effectively drive downstream neurons [1]–[4]. To date, our understanding of synchrony-based neuronal routing has been dominated by two models which attribute the origin of synchrony to dissimilar mechanisms.

According to the first model, synchronous spiking activity is both created and routed through dense and/or strong convergent-divergent connections between subsequent layers of feedforward networks (FFNs). In this scenario, these connections are a source for shared and correlated input that provides sufficient synchronization for spiking activity to propagate across the FFN [5]–[10].

However, the requirements of either strong synapses or high connection probability pose serious constraints on the biological plausibility of these FFNs in the cortex, in which connectivity is in general sparse [11] and synapses are weak [3], [9], [12]. Even though, the sparse cortical connectivity could in theory host a large number of sparsely and weakly connected (diluted) FFNs, they would fail to generate enough synchronization to ensure propagation of spiking activity [7], [13], [14].

The second model suggests that population oscillations could soften the requirement of strong/dense connectivity by enhancing synchronization and neuronal excitability during the excitable phase of the oscillation [15], [16]. A key requirement for this propagation mode is that oscillations, which are generated locally due to interactions between excitatory and inhibitory neurons, must maintain a consistent phase relationship (coherence) between the communicating networks (“communication through coherence”; [15], [17], [18]). However, the mechanisms underlying the generation and maintenance of such coherent oscillations between distant brain areas have remained elusive despite a number of theoretical proposals [19].

Here, we propose a novel mechanism by which oscillatory activity exploits the presence of resonance frequencies in networks of excitatory and inhibitory neurons (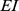) to promote the propagation of synchronous activity across diluted FFNs (“communication through resonance”). The role of such network resonance is to amplify weak signals that would otherwise fail to propagate. According to our model, coherent oscillations emerge in the network during slow propagation of synchrony, while at the same time synchrony needs these oscillations to be propagated. Thus, spreading synchrony both generates oscillations and renders them coherent across different processing stages. This abolishes the requirement for separate mechanisms providing the local generation of oscillations and establishing their long-range coherence. Moreover, coherence between oscillations may be viewed as a consequence of propagation instead of being instrumental to establish communication through synchrony. Our results also suggest that the emergence of coherent oscillations is influenced by the dynamical state of the ongoing activity. We propose that changes in the ongoing activity state can have an influence on cortical processing by altering the communication between different brain areas.

## Materials and Methods

### Network model

**Figure 1:**
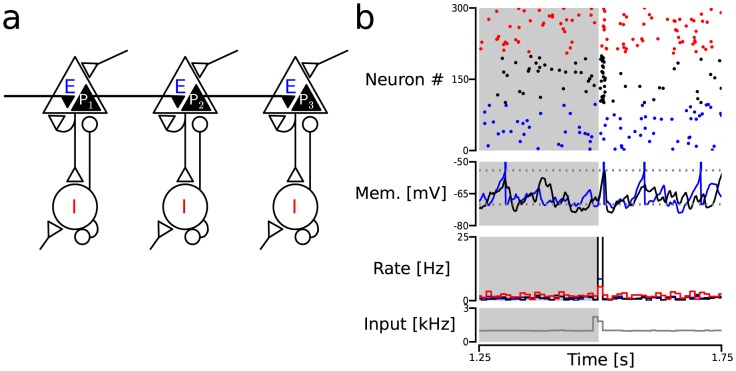
Architecture and ongoing activity of the diluted FFN model. (**a**) Scheme of a 3-layer FFN. The color code is preserved across figures: red/blue/black represent 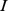/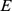/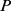 neurons. (**b**) Pulse packet response in an isolated layer. A pulse packet (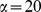; 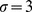) was presented after 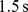. Gray shaded rectangle: ongoing activity region. Subpanels: raster plot of the spiking activity (top); membrane potential traces of two example 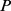 neurons (upper-middle); output rate histogram (lower-middle); and input rate histogram (bottom).

**Figure 2:**
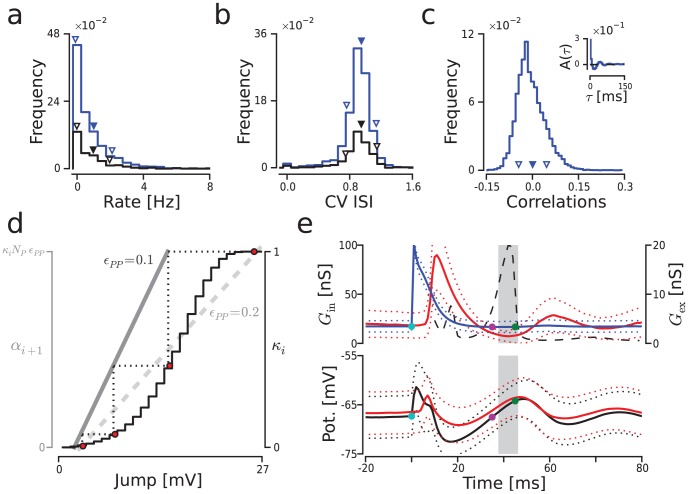
Ongoing dynamics and impulse response of an isolated FFN layer. (**a–c**) Ongoing activity statistics computed in absence of pulse packet stimulation (gray region in Figure 1b) from a simulation of 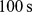. Color code as in Figure 1. Filled/empty inverted triangles: mean/s.d.. (**a**) Mean rate distribution of individual neurons. (**b**) Distribution of 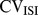 values. (**c**) Distribution of pairwise correlation coefficients (inset: auto-covariance function of the population spike train). (**d**) Pulse packet amplitude transfer map. Black trace: membrane potential distribution of 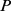 neurons in its c.d.f. form plotted as a function of the distance to spike threshold (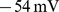; “Jump”). Gray lines: average voltage depolarization (“Jump”) caused by a pulse packet of 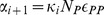. Dark gray line: depolarization when 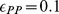 which is the value used in this work. Light gray line: depolarization when 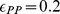. Red dots and dotted lines: trajectory of a pulse starting from a fully activated layer (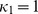). (**e**) Effect of stimulation with a single pulse packet (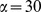; 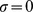). Subpanels: time evolution of inhibitory and excitatory conductances (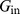 and 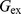) averaged across 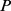 neurons (upper); and evolution of the membrane potential distribution for 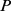 and 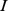 neurons (lower). Gray region: optimal time window for the arrival of a hypothetical second pulse packet. Cyan dot: arrival time of the actual pulse packet. Magenta/green dot: hypothetical arrival of a second pulse outside/inside of the optimal time window. Dotted lines: mean 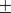 s.d. across neurons. Black dashed line: Same as blue trace in Figure 4b bottom (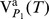), resonance curve plotted as a function of time interval instead of frequency.

**Figure 3:**
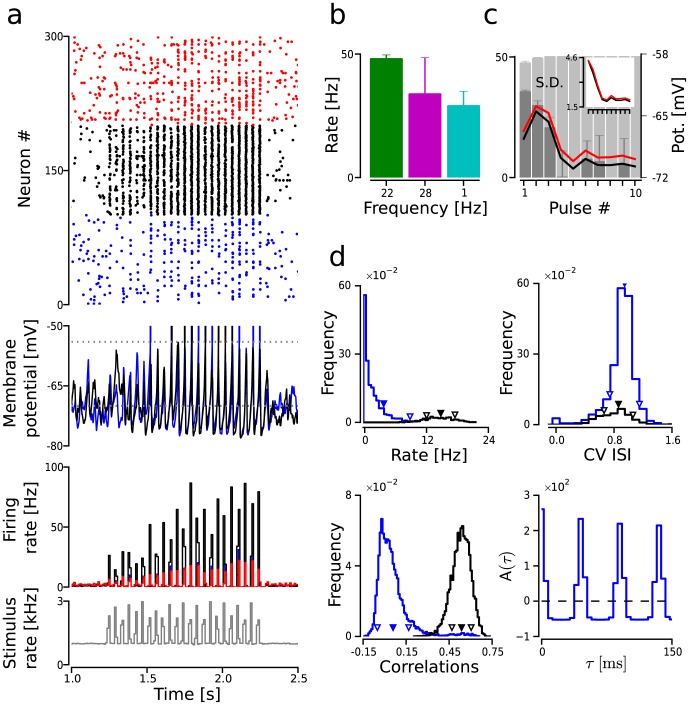
Resonance in an isolated FFN layer. (**a**) Network activity during stimulation with a train of pulse packets (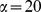; 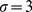; 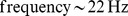). Color code as in Figure 1. Subpanels: raster plot of the spiking activity (top); membrane potential traces of two example 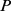 neurons (upper-middle); output rate histogram (lower-middle); and input (to 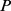 neurons) rate histogram (bottom). (**b**) Increased mean firing rate at the layer's resonance frequency within 20 ms after the pulse packet arrival. 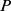 neurons were stimulated with trains of periodic pulses packets (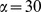; 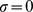; 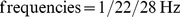). Error bars: average s.d. across trials (**c**) Increased activation caused by dis-inhibition. Red/black line: average mean of the membrane potential distribution of 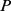/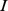 neurons sampled 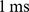 prior to the arrival of the pulse. Train of pulses as in b with frequency 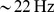. Inset: average s.d. of the membrane potential distribution across neurons. Light gray bars: 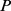 rate response calculated as in (b). Dark gray bars: 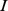 firing rate within 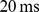 before the pulse packet arrival. (**d**) Spiking and membrane potential statistics measured during 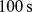 of stimulation. Stimulus statistics as in (c). Subpanels: distribution of individual mean firing rates in Hz (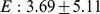; 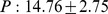; mean 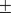 s.d. across population; upper left); distribution of 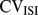 (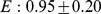; 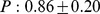; upper right); distribution of pairwise correlation coefficients (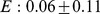; 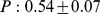; lower left); and auto-covariance function of the population spike train (lower right).

The network models were multi-layered FFNs. Each layer consisted of two recurrently connected homogeneous neuronal populations. In **Figure 1**, **Figure 2** and **Figure 3** we used 2,000 excitatory (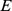) and 500 inhibitory (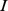) neurons. For the rest of the figures, we reduced the number of 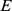 neurons to 1,000 while keeping the number of interlayer projecting neurons fixed to 300. This reduction, which was done in order to improve simulation efficiency, did not affect the results in any qualitative manner.

The connectivity within each layer was random with the following connection probabilities: 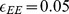 and 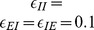, where 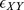 denotes the probability of connection from a neuron in population 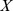 to a neuron in the population 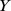. Connections between layers were strictly feedforward and excitatory, and restricted to a sub-population of 300 randomly-chosen 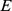 neurons (in the rest of the paper referred to as 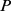) in every layer. The interlayer connectivity was sparse with probability 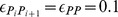 (cf. **Table 1**).

**Table 1:** Network parameters.

Name	Value	Description
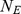	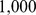	Size of excitatory population
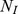	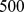	Size of inhibitory population
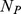	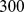	Size of the group of projecting neurons
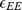	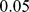	Connection probability from excitatory to excitatory
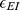	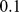	Connection probability from excitatory to inhibitory
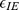	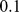	Connection probability from inhibitory to excitatory
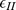	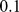	Connection probability from inhibitory to inhibitory
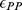	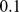	Connection between projecting neurons from different layers

### Neuron model

Neurons were modeled as leaky integrate-and-fire neurons, with the following membrane potential sub-threshold dynamics:
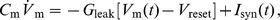
where 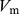 is the neuron's membrane potential, 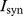 is the total synaptic input current, 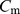 and 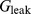 are the membrane capacitance and leak conductance respectively. When the 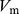 reached a fixed threshold 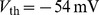 a spike was emitted and the membrane potential was reset to 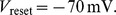 After the reset, the neuron's membrane potential remained clamped to 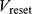 during a time period 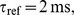 mimicking the period of absolute refractoriness. All other parameters are detailed in **Table 2**.

**Table 2:** Neuron parameters.

Name	Value	Description
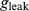	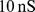	Membrane leak conductance
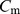	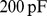	Membrane capacitance
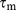	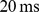	Resting membrane time constant
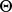	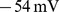	Fixed firing threshold
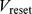	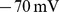	Reset potential
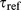	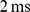	Absolute refractory period

### Synapse model

Synaptic inputs consisted of transient conductance changes:
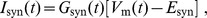
where 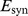 is the synapse reversal potential. Conductance changes were modeled using exponential functions with 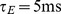 and 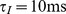.

**Figure 4:**
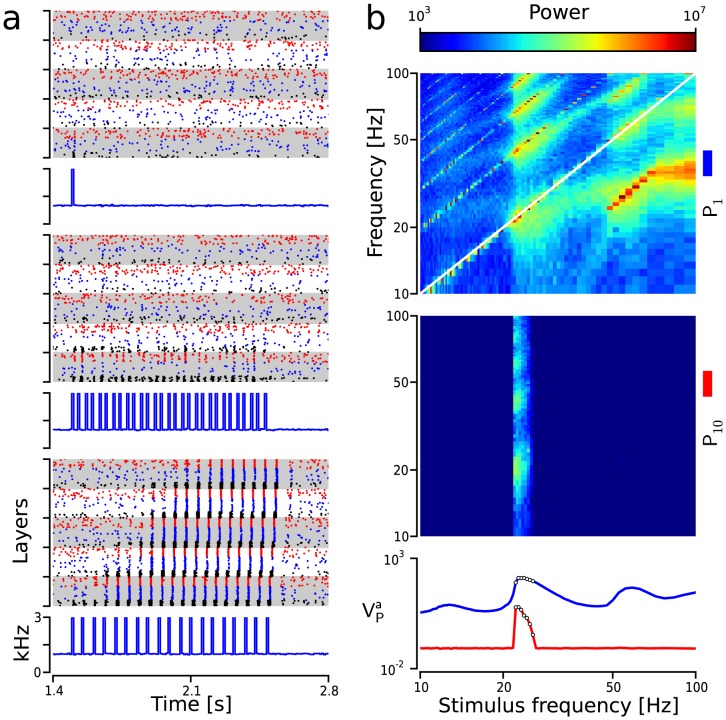
Communication through resonance in diluted FFNs. (**a**) Example simulations illustrate the transmission of synchrony in 5-layer diluted FFNs for three different stimulus frequencies: 1 Hz (top), 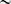 28 Hz (middle) and 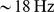 (bottom; resonance frequency for this network). Pulse packets: 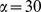 and 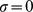. In all three subpanels: stimulus time histogram in kHz (bottom) and raster plot of spiking activity (top). Gray/white stripes: different layers. Color code as in Figure 1. (**b**) Propagation of synchronous activity in 10-layer FFNs as a function of the stimulus frequency (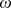). Activity of the first layer (blue trace in bottom subpanel and top subpanel) and the last layer (red trace in bottom subpanel and middle subpanel) during periodic stimulation at different frequencies (pulses: 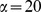 and 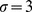). Top/middle subpanels: 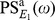/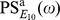. Bottom subpanel: 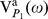 (blue trace) and 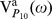 (red trace). White circles: 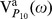 significantly larger than 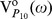. Note that 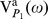 was previously depicted in Figure 2e as a function of the inter-pulse interval 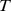 for comparison with the average network response to an isolated synchronous pulse.

**Figure 5:**
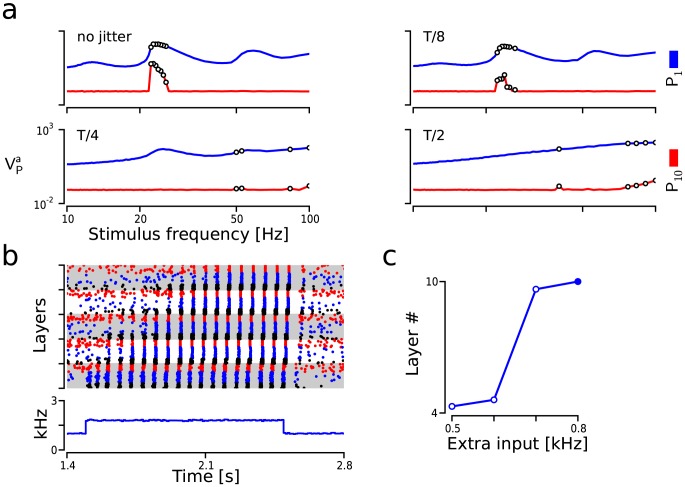
Robustness of CTR against deviations from periodicity. (**a**) Effect of jittered arrival times on CTR. Resonance curves (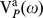) of 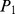/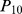 (blue/red) as a function of the amount of jitter. Pulse packets: 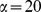 and 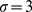. Jitter is expressed as a fraction of the input's interval 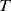. White circles: 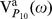 significantly larger than 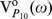. (**b**) Non-periodic Poisson input to 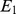 neurons in a 5-layer FFN triggers CTR. Subpanels: Raster plot of the spiking activity (top); and input (to 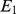 only) rate histogram in kHz (bottom). Color code as in Figure 1. (**c**) Last layer reached by the propagating synchronous activity as a function of the additional input rate to 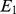. Solid circle: stimulus as in b.

Synaptic delays were set to 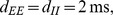 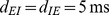 and 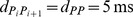 in **Figure 4a**, **Figure 5b–c** and **Figure S4a**. In the rest of the figures, delays were set to 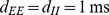, 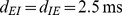 and 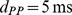. Longer delays produced a stronger and more reliable propagation and therefore were chosen to illustrate the propagation across layers in **Figure 4a**. The choice of delays influenced the resonance properties of the network [20]. However, the general principle remained unaffected. Other parameters are detailed in **Table 3**.

**Table 3:** Synapse parameters.

Name	Value	Description
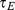	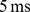	Rise time of excitatory conductance
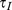	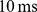	Rise time of inhibitory conductance
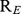	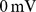	Reversal potential of excitatory synapses
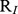	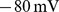	Reversal potential of inhibitory synapses
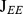	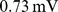	At a holding potential of 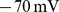
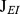	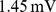	At a holding potential of 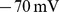
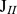	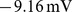	At a holding potential of 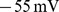
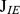	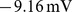	At a holding potential of 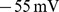
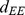	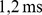	delay of excitatory to excitatory synapses
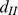	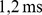	delay of inhibitory to inhibitory synapses
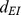	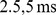	delay of excitatory to inhibitory synapses
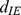	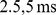	delay of inhibitory to excitatory synapses
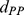	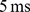	delay of interlayer synapses

### External input

**Figure 6:**
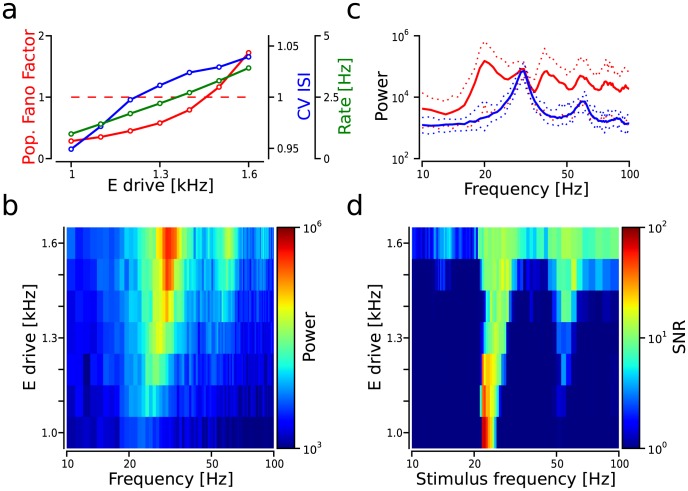
Effect of the dynamical network state on CTR. (**a**) Population spiking statistics as a function of 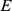 drive. Blue line: mean 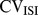; red line: population Fano factor (pFF); and green line: mean firing rate. Dashed red line: separation between synchronous and asynchronous state based on the pFF. (**b**) Effect of 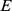 drive on 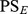. (**c**) 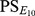 with 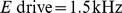. Blue/Red trace: average 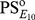/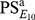; dotted traces: maximum (upper) and minimum (lower) power values computed across input frequencies for each case. (**d**) SNR measured in 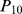 as a function of 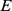 drive.

Each neuron was driven by 1,000 independent Poisson excitatory spike trains with an average rate of 1 Hz each (i.e., a total average input rate of 1 kHz), which mimicked uncorrelated background inputs coming from other brain areas. In **Figure 6**, 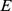 neurons received this external drive (referred to as 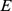 drive) with larger rates than 1 kHz as indicated in the figure.

The synchronous stimuli consisted of periodic trains of synchronous spikes (pulse packets) with different frequencies. Only 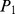 neurons received these additional spikes. The individual pulse packets consisted of a fixed number (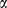) of spikes per neuron, distributed randomly around an arrival time 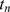. The time of each individual spike was drawn independently from a Gaussian probability distribution centered around 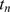 and with s.d. (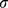). In **Figure 2e**, **Figure 3b–c**, **Figure 4a**, 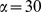 spikes and 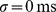 (i.e, perfectly synchronous). In the remaining cases 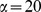 spikes and 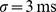.

When the stimulus was a periodic train of pulse packets, we set the frequency of stimulation by adjusting the period (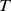) between arrival times 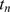 (i.e., the center of the Gaussian p.d.f.). When 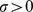, the spikes were spread around 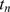, as indicated above, and therefore the time distance between the last spike from a given pulse packet and the first spike from the next was always variable for the same input frequency. The smallest interval that was used between arrival times was 10 ms (100 Hz) and the largest 100 ms (10 Hz). Additionally, in **Figure 3b** we used 1 Hz stimulation.

In simulations where the arrival times were jittered, the size of the jitter was drawn from a uniform distribution centered on the arrival time 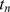. The extent of the jitter window was chosen to be a function of the interval 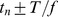, where 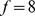, 4 or 2 in order to make the effect comparable across different frequencies.

### Data analysis

To compute the auto-covariance functions 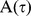 (inset in **Figure 2c** and **Figure 3c** bottom right; only positive time lags are shown), time was divided into bins of 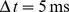 and the population spike trains were transformed into spike count vectors 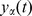, where 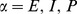 denotes the population. The auto-covariance functions were then computed as follows:
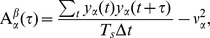
where 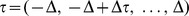, 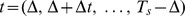, 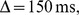 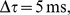 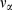 indicates the population mean firing rate and the superscript 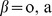 denotes *ongoing* (computed from a single 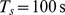 simulation in absence of pulse packet stimulation) and *activated* (computed from 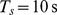 of activity during stimulation starting 5 s after the stimulus onset and averaged across 20 trials), respectively.

We used the population Fano factor (pFF) to classify the population spiking activity states as synchronous or asynchronous (dashed line in **Figure 6a**). We used the central value of 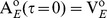 (variance) normalized by the mean population firing rate:
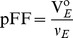



The signal-to-noise ratio (SNR) in **Figure 6d** was computed as follows:
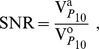
where 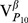 indicates the variance of the spiking activity of 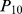 neurons as indicated above.

Pairwise correlations were computed using the Pearson correlation coefficient between the spike count vectors of pairs of neurons (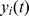 and 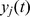).
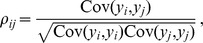
where:



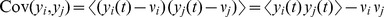
and 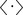 indicates time average and vectors 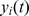 and 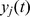 were computed using a time window of 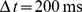. We used 10,000 pairs to compute the distributions shown in **Figure 2c** and **Figure 3d**. The correlation coefficients were computed from simulations with a length of 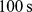.

The power spectrum of the population spike train (PS) was calculated as follows (from [21]):
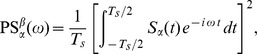
where, 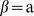 in **Figure 4b**, **Figure S4a** and **Figure S5a** and 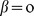 in **Figure 6b** indicating the corresponding value of 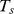 (cf. description of the auto-covariance function above).

### Simulation tools

Network simulations were performed using the simulator NEST, interfaced with PyNest [22], [23]. The differential equations were integrated using forth order Runga-Kutta with a time step of 0.1 ms. Simulation data was analyzed using the Python scientific libraries: SciPy and NumPy. The visualization of the results was done using the library Matplotlib [24]. The code to reproduce several results presented in this work (**Figure 1b**, **Figure 3a**, **Figure 4a**, **Figure 5b** and **Figure S6a**) is available at https://github.com/AlexBujan/ctr. Other results can be reproduced by modifying that code.

## Results

### Network model: Diluted FFNs

We studied the propagation of synchronous spiking activity across diluted FFNs with sparse interlayer connectivity. In this model, each layer represented a small neocortical network with 2,000 excitatory (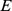) and 500 inhibitory (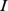) neurons. The connectivity within each layer was sparse and random. The connections between layers, which modeled long-range projections between different cortical networks, were strictly feedforward and excitatory. These interlayer projections were restricted to a sub-population of 300 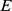 neurons which we refer to as *projecting* neurons or 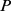 neurons throughout the manuscript (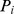 refers to all the projecting neurons in a layer with the subscript 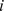 indicating the position of the layer in the FFN; cf. **Figure 1a**). Interlayer connection probability 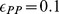 and hence, each 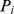 neuron received, on average, 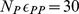 connections from the previous layer (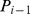; cf. **Table 1**).

All layers were driven by external Poisson input spike trains and the synaptic weights were adjusted (cf. **Table 3**) to bring the network into an asynchronous-irregular (AI) activity regime [25], [26], consistent with the statistics of cortical activity in awake behaving animals [27]–[30]. The mean firing rate of individual excitatory neurons showed a heavy tailed distribution with a mean of 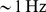 (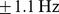; 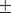 s.d. across the population). The mean coefficient of variation of the inter-spike interval distribution (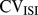) was 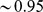 (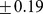) and the distribution of pairwise correlations was centered around zero with a mean of 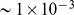 (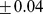) (cf. **Figure 1b** and **Figure 2a–c**). The activity of the 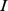 population was also irregular and asynchronous although with slightly higher mean firing rates (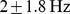). These results were computed from a single simulation of 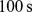 duration.

To study the propagation of synchrony, we stimulated all 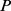 neurons in the first layer (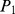) with synchronous events or pulse packets (cf. Methods). The synaptic strength of these input synapses was equivalent to the other 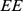 synapses in the FFN (cf. **Table 3**).

First, we checked that the connectivity between layers was indeed too weak to support the propagation of single synchronous events. To this end, we generated an *amplitude transfer map* which we used to estimate the change in amplitude undergone by pulse packets as they travel across the FFN. This map, shown in **Figure 2d**, was generated using the ongoing membrane potential distribution (black trace) and depolarization transfer function (dark gray solid trace) of the 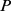 population. The measured membrane potential distribution (computed from 100 s of ongoing activity) is shown as the cumulative density function (c.d.f.) of the distance to threshold (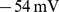). When represented as such, the probability of being at a certain distance from threshold can be interpreted as the fraction of cells (here named 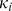, where 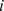 indicates layer index) that will spike if a depolarization (“jump”) equivalent to such distance is applied to all 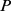 cells. The membrane potential transfer function was calculated by measuring the averaged maximum depolarization across 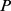 neurons induced by a pulse of perfectly synchronous spikes (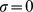) with different amplitudes 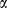. The mapping between the two curves can be done by knowing the relationship between the activation level of the 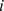
th layer 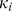 and the amplitude of the pulse packet received by the subsequent layer 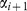 which in this case is as follows: 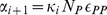. Knowing this relationship, it is then possible to project a point from one curve to the other, thereby drawing an estimated trajectory of the pulse packet's amplitude across the chain. In the figure, an example of such a trajectory is illustrated with red dots and dotted lines. To make a convincing case, we started the trajectory with a fully activated first layer (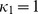; upper red dot) and followed the pulse packet until it reached a stable point (intersection between the two curves). Such trajectories will always end at an intersection between the curves which in this case (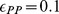) is found only at zero. This shows that any single pulse traveling across this FFN will eventually vanish, regardless of the initial value of 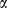. Similarly, it can be shown that if the connectivity is raised to 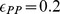 (dashed light gray line) a single pulse can undergo a stable propagation for some initial values.

### Resonance in recurrent EI networks

After a perturbation caused by a synchronous pulse, the network's activity relaxed back to ongoing levels while displaying a stereotypical damped oscillation (**Figure 2e**). This dynamics, which was observed both at the spiking level (shown as conductances in 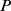 neurons in **Figure 2e** top) and the level of the membrane potential (**Figure 2e** bottom), indicated that the network had resonance frequencies. The presence of such resonance frequencies suggested that stimulating the network with a periodic train of pulse packets, within a specific frequency range, could induce a large response even for weak stimuli (e.g., pulse packets consisting of a few weakly synchronized spikes).

The existence of resonance behavior in 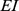 networks has already been shown elsewhere [20]. Here, we analyzed the network response to a pulse packet stimulation in order to understand in more detail how resonant dynamics can emerge in these networks. During the transient damped oscillatory response, there was a brief time period of a few milliseconds (indicated approximately as a gray region in **Figure 2e**) during which 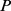 neurons were slightly more depolarized (higher mean), more synchronous (decreased s.d.) and their inhibitory conductance was reduced. This suggested that the arrival of a second pulse packet inside this brief time window (e.g., around 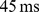 after the arrival of the first pulse packet; shown as a green dot in **Figure 2e**) should result in a larger activation as compared to the first pulse. Conversely, the arrival of a second pulse outside of this window (magenta dot in **Figure 2e**) would only lead to a similar or even weaker activation.

To confirm this, we stimulated 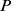 neurons in an isolated layer with a sequence of 100 periodic pulse packets (identical to the ones described in the previous section; 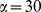 and 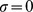) and computed the mean firing rate within 20 ms after the arrival of each synchronous event (which was found to be an appropriate time window to capture the pulse packet induced modulation of the firing rate). We repeated the experiment using three different time intervals 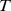: 35, 45 and 1,000 ms (**Figure 3b**) and in each case the results were averaged across 100 trials. Pulse packets separated by 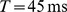, which matched the optimal window described above, resulted in an average spiking activity of 48 Hz (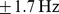, 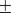 s.d. across trials; green bar in **Figure 3b**). By contrast, stimulation with pulse packets separated by 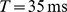 could only induce a mean network response of 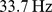 (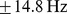). This response was comparable to a stimulation in which pulse packets arrived at an interval of one second, long after the transient response to each individual event had died out (compare magenta and blue bars in **Figure 3b**). This result confirmed that a train of periodic pulses, with a period adjusted to match the optimal time window, was able to elicit a stronger response as opposed to a single pulse packet. Additionally, the fact that a higher input frequency resulted in a lesser activation suggested that this effect was not merely due to the temporal integration of the individual pulse packets.

To further understand the emergence of resonance in these networks, we analyzed the temporal evolution of the membrane potential distribution (mean and s.d. sampled 1 ms prior to the arrival of each pulse packet) during stimulation with a train of 100 pulse packets separated by 45 ms (**Figure 3c**). The results were averaged across 100 trials. A brief initial depolarization, caused by the first two pulse packets, was followed by a sustained hyper-polarization in both 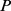 and 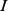 neurons as more pulse packets were presented. The hyper-polarization reflected that a larger fraction of neurons was refractory (or close to the spike reset potential) due to the increase in firing rate (light gray bars in **Figure 3c**) and recurrent inhibition. The fact that most neurons were more hyper-polarized seemed to be at odds with the observation that the pulse packets were more effective in driving 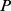 neurons. Furthermore, a decrease of the s.d. (**Figure 3c** inset) indicated that 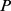 neurons were overall more synchronized, namely, that the hyper-polarization was shared across the entire population. Essentially, the increased responsiveness was a consequence of the fact that 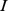 neurons were effectively refractory at the time of the arrival of pulse packets, as indicated by the progressive reduction in their firing rates (**Figure 3c** dark gray bars). That is, although 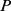 neurons moved farther away from the spiking threshold, they received less inhibition at the time of the arrival of the pulse packets which resulted in stronger activation. This observation hinted to an important role of 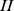 connections in the emergence of resonance in these networks.

We investigated the contribution of the 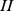 loop to the generation of resonance by conducting simulations in which we progressively reduced the strength of the recurrent inhibitory connections (**Figure S1**). We compensated the reduction in 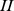 input by adding an additional source of external inhibitory conductance in order to keep the firing rate of the 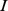 neurons (measured during the ongoing state) constant across conditions. Our results showed that although the 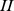 loop had a substantial effect on the resonance peak's amplitude and frequency, the network still had resonant properties in the absence of an 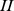 loop. This indicates that while 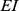 connections are sufficient to create resonance, 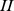 dynamics play a facilitating role. In addition to the hyperpolarizing inhibition used in our model, other biologically plausible mechanisms, such as shunting inhibition or gap junctions, could also enhance resonance [31]–[33].

Although the overall activity of an isolated layer became more synchronized, with network oscillations that were locked to the stimulus, the overall activity of 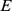 neurons remained fairly irregular (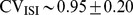), and mean pairwise correlations were still relatively low (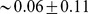; compare (**Figure 3d** and **Figure 2c**). Hence, the activity of 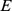 neurons during stimulation was still consistent with biological data, which shows that cortical firing is highly irregular despite the presence of oscillations at the population level as measured by local field potentials [34], [35]. Note however that the activity of the 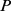 neurons was more regular (they skipped fewer cycles) than the other 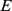 neurons. Such a level of regularity in the 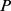 population was needed in order to induce oscillations in the post-synaptic layer and was a consequence of the small number of 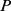 neurons together with the sparse inter-layer connectivity. Thus, the choice of a larger 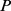 population size and/or a higher connection probability could make propagation compatible with a more irregular firing in the projecting population (cf. below).

Additionally, we explored whether our network model operated in a linear regime in which case the tools of linear systems analysis could be applied to further understand the resonance [20]. To this end, we calculated the amplitude of the network's response when stimulated with synchronous pulses for different values of 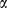. Our results indicated that the behavior of the simulated network was generally non-linear showing a saturation of the response amplitude with high 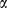 and a progressive shift in the resonance frequency (**Figure S2**). However, we also found that within a restricted range of input amplitudes the network's response approached linearity (cf. straight lines in **Figure S2e**).

### Communication of synchrony through network resonance

Next, we addressed the question whether the network resonance-induced amplification of stimulus responses, observed in isolated layers, could be sufficient to enable the transmission of synchrony in diluted FFNs, which did not support the propagation of individual pulse packets.

To this end, we stimulated a 5-layer FFN with three different frequencies, that were analogous to the ones introduced in the previous section (cf. Methods). The amplification, observed when the input frequency matched the resonance frequency of 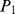, proved to be sufficient to induce a successful transmission across the entire FFN (**Figure 4a** bottom). As expected, when the stimulus had a different frequency from the resonance frequency, or it consisted of a single pulse packet, the synchronous activity did not reach the last layer (**Figure 4a** top and middle). Since the transmission relies on the network resonance, we refer to this mode of synchronous activity propagation as “communication through resonance” (CTR).

After receiving a few input cycles at the resonance frequency, nearly all 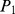 neurons started to fire near synchronously every time a new pulse was presented. At this point, even though a large number of synchronous spikes were produced in the first layer, the sparse interlayer connectivity (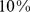) reduced this increased activation to a train of weak pulse packets with an average of 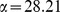 spikes (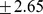 spikes) and 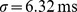 (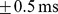), which prevented the propagation of synchronous volleys immediately after amplification had taken place in 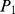. Therefore, amplification through resonance was needed at every layer to propagate the activity across the FFN due to the diluted interlayer connectivity.

Next we investigated how the frequency of stimulation affected the propagation of synchrony across a 10-layer diluted FFN. Expectedly, we found a correlation between resonance-induced increase in synchrony in 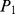 and the successful communication of synchronous events across the entire FFN (**Figure 4b**). To quantify the synchrony we calculated the variance of the 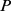 population spike train (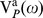 where 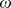 indicates the stimulus frequency; cf. Methods). We then used 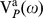 to construct resonance curves as shown in **Figure 4b** bottom. A propagation was labeled as successful when 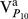 was significantly increased (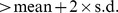; white dots in **Figure 4b** bottom) with respect to the baseline value 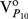. The spectral analysis of the spiking activity revealed that the increase in power in the last layer was always more pronounced at 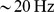, which was approximately the resonance frequency of the network (cf. **Figure 4b** lower-middle subpanel). Furthermore, CTR was not restricted to the FFN architecture discussed thus far. Our results showed that at least two alternative interlayer connectivity patterns also supported CTR: when receiving neurons were restricted to a specific sub-population of 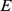 neurons but any 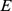 neuron could project to the next layer (**Figure S3a**); when any 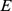 neuron could receive and send projections (**Figure S3b**).

However, even when neuronal activity propagated to the last layer (white dots in **Figure 4b**), 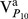 was significantly lower than in 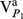 (compare red and blue curves in **Figure 4b** bottom). This result indicated that propagation was occasionally characterized by failures of synchronization of the last layers. Thus, the ratio 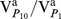 could be used as a proxy for the propagation reliability when activity was observed during long time periods (10 s). Generally, networks that produce a moderate amplification of the signal at the resonance frequencies would be more sensitive to noise fluctuations, which can transiently reduce the degree of synchrony and lead to frequent propagation failures. A larger amplification, which in our model was achieved by introducing longer delays within each layer, lead to a perfectly reliable propagation at the resonance frequencies (**Figure S4a**).

For the parameters used here, the range of frequencies that led to a successful propagation approximately spanned from 22 to 26 Hz. The extent of this frequency range can be varied by an appropriate choice of network parameters (cf. **Figure S4a**; see [20] for a more detailed study on the effect of different parameters on resonance). The effect of different parameters on the resonance profile of the network can be estimated using the network's average response to a single pulse packet stimulation (cf. **Figure 2e**). When the input frequency is expressed as the time interval between pulse packets 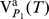 (dashed black trace in **Figure 2e** top), the resonance profile can be related to the average network response. Note that 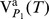 is again represented as a function of the input frequency 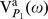 in **Figure 4b** (blue trace). As can be seen in **Figure 2e**, the dominant peak in 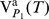 closely matches the trough of the average inhibitory conductance response (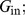 red curve). This suggests that the network's response to a single pulse packet stimulation can predict its resonance curve and thus can be used to understand how different changes in the network parameters may affect the resonance properties of the network. While different network parameters can alter its resonance curve, the activity propagation based on network resonance would remain essentially the same.

For this specific choice of parameters, 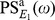 (**Figure 4b** top; cf. Methods) revealed that the resonance occurred mainly around two main stimulus frequencies: 23 Hz and 58 Hz (see also **Figure S5b** bottom row). Note that similar resonance frequencies were found when neurons were stimulated with a sinusoidally modulated Poisson input, which indicates that the faster resonance frequency can not be explained by the existence of harmonics of the base frequency present in the periodic input pulse train (**Figure S6**). Naturally, the smaller resonance frequency precisely matched the time window described in the previous section. The frequency of the second resonance peak can be explained using the network's average response as indicated earlier. To understand this effect, we can consider a simpler stimulus consisting of three pulses the frequency of which is systematically increased with respect to the main resonance frequency (23 Hz). Initially, the rise in frequency will cause the second and third pulses to arrive outside the optimal time window. However, as the frequency is further increased, a frequency will be reached for which the third pulse will fall inside the optimal window giving rise to an increase of the spiking response. Intuitively, this latter frequency should be approximately twice as large as the main resonance frequency, which is inconsistent with our results. This discrepancy can be understood when we notice that the second pulse, although not strong enough to activate 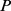 neurons, does accelerate their re-polarization, thereby advancing the optimal time window within which the third pulse should arrive. That is, the subthreshold effect of these incommensurate pulses will speed up the network response resulting in the second resonance peak being faster than twice the main resonance frequency.

### Deviations from periodicity and sustained input signals

Experimental evidence suggests that brain oscillations in the gamma range are not perfect periodic oscillators with a consistent phase [36]–[39]. Consequently, to be a biologically plausible mode of communication, CTR should be robust enough to facilitate the transmission of oscillatory spiking activity when the constraint of a constant phase has been relaxed.

To quantify the extent to which CTR could afford unstable phases within an oscillation, we probed 10-layer diluted FFNs with periodic trains of pulse packets whose arrival times were jittered. The jitter was drawn from a uniform distribution centered on the arrival time (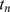) of the pulse packet. The extent of the jittering window was chosen to be a function of the interval 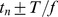 where 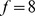, 4 or 2. The results showed that CTR could still enable the transmission in the presence of moderate amounts of jitter (**Figure 5a**). For this particular selection of network parameters, a jitter of 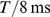 did not alter the main characteristics of the amplification process and the activity propagated to the last layer (**Figure 5a** top right).

However, if the jitter was further increased the activity propagated to fewer layers and the propagation was more unreliable. Interestingly, for a jitter of 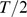, which corresponds to completely aperiodic pulse packet train, we observed that activity propagation increased with increasing the stimulus frequency. However, in this case also the pulse packets propagated with a frequency of 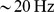, close to that of the network resonance frequency (**Figure S4b**). That is, each FFN layer acted like a bandpass filter, which suggested that a broad-band noise stimulus could also trigger the transmission since it can generate oscillations close to the resonance frequency.

Indeed, it is well known that the dynamics of 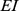 networks can display oscillations at the population level when they are stimulated with strong unstructured external drive [26], [40]. We hypothesized that in the FNN a constant rate Poisson input could bring the activity of the first layer into an oscillatory regime, thereby generating a train of weak pulse packets that provide rhythmic input to the subsequent layers.

We tested this hypothesis by replacing the oscillatory input to 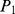 by an additional source of constant Poisson input to all 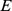 neurons in the first layer. When in the network shown in **Figure 5b-c** the 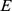 drive was increased from 1 to 1.8 kHz the activity became oscillatory with enough power to ignite the resonance in the second layer. Interestingly, the frequency of the oscillations in 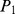 was comparable to the resonance frequency of the network. This is not surprising as both resonance and oscillations at higher input regimes are shaped by the same network time constants, e.g., synaptic delays and membrane time constants [20].

Thus, we show that both slightly phase-jittered oscillatory inputs at the resonance frequency and broad-band stimulation are compatible with CTR in diluted FFNs.

### Effect of the network state

Thus far, we have assumed that ongoing activity in each individual layer of the FFN was AI with low firing rates. However, there is ample experimental evidence suggesting that cortical networks *in vivo* can display more synchronized ongoing activity regimes depending on the behavioral state of the animal [30], [41]. We therefore explored how the propagation of pulse packets via CTR is influenced by the dynamical state of the spontaneous network activity.

The level of synchrony in recurrent 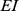 networks can be modulated by adjusting the firing rate of the external excitatory input [9], [26], [42]. Here, we changed the dynamical state by increasing the 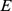 drive from 1 to 1.6 Hz. Lower rate 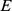 drive gave rise to very sparse and asynchronous firing patterns, which progressively became more synchronous as the 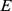 drive was increased (synchrony measured as population Fano factor; red line in **Figure 6a**). The spiking activity of individual neurons remained irregular (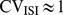) for the parameter space explored here (cf. blue line in **Figure 6a**). 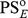 increased in the range between 10 and 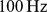 for larger values of 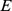 drive. This increase was more pronounced around the peaks, which progressively shifted towards faster frequencies as the external input became stronger (**Figure 6b**).

To study the effect of network synchrony on CTR, we stimulated 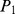 in 10-layer FFNs with periodic trains of pulse packets for the different levels of 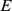 drive and computed the signal-to-noise ratio (SNR) in 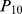 (cf. Methods). Generally, more synchronized activity states enabled CTR within a broader range of input frequencies, however the largest SNR values in 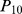 were found at the low input regimes (**Figure 6d**). Independent of the synchrony level, resonance frequency and subsequently CTR were always confined within a range of input frequencies that closely matched the frequency around the peaks of 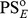 (compare **Figure 6d** and **Figure 6b**). Hence, the resonance frequencies also became faster at higher levels of 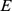 drive. This shift reflected the reduction of the time that neurons needed to recover from the effective refractory state (absolute refractory period and hyper-polarization time) due to the presence of larger amounts of excitation as 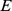 drive was increased. The main peak in 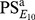 when activity reached 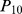 was invariably found at 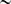 20 Hz. This value was slower than the mean peak measured in 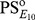 which was 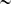 28 Hz (**Figure 6c**). The values of 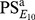 were larger than those of 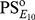 for all the frequencies analyzed here. Notably, this difference was more pronounced in the gamma range (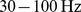) as compared to lower frequencies (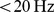; cf. **Figure 6c**).

Interestingly, network synchrony improved the propagation for faster input oscillatory regimes (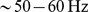, **Figure 6d**). In summary, our results showed that the ongoing state had opposite effects on CTR depending on the input frequency range. For lower input frequencies, AI activity increased SNR, while for larger input frequencies SI could enable the propagation which was absent during AI.

### Model validation and implications for population coding

A direct validation of the model will involve the induction of coherent oscillations between distant brain areas by stimulating excitatory neurons in the presynaptic area at the resonance frequency. The resonance profile of a neuronal population can be obtained by recording its activity during periodic stimulation of the neurons with different frequencies. Similar experiments, which made use of optogenetic tools, have already been performed to study the role of specific cell types in the generation of gamma oscillations [43]. According to our model, even weakly connected distant networks (verified, e.g., by anatomical or electrophysiological studies) with a similar resonance profile can engage in a coherent oscillation by stimulating the presynaptic population at the resonance frequency. In contrast, a stimulation protocol, which does not induce a strong oscillation in the stimulated area, will fail to form such a coherent activity with the distant population. Our model also predicts a progressive entrainment characterized by a gradual increase in the measured power over multiple stimulation cycles in the stimulated presynaptic network. A similar entrainment should be found in the postsynaptic network with a certain delay which should be a function of the connectivity strength (see discussion). Moreover, in CTR mode of propagation the oscillations emerge only after a delay and not directly at the onset of the stimulus. This feature of the model is consistent with the observation that 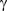 - band oscillations appear after 100 ms of the stimulus onset (e.g. [44]).

**Figure 7:**
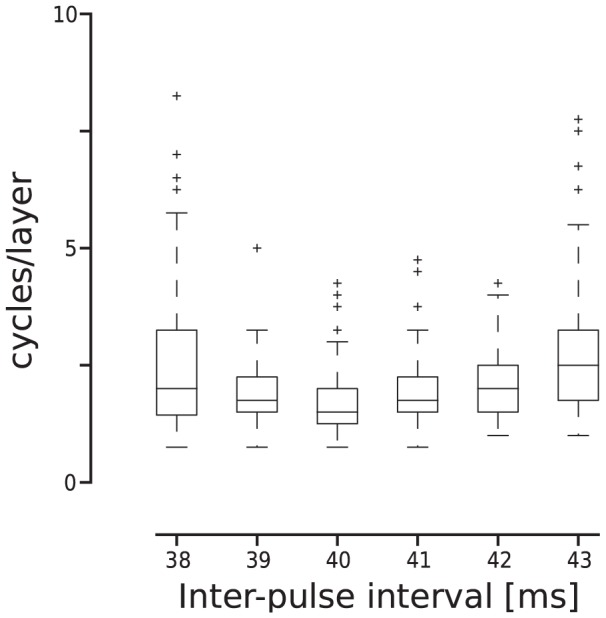
Propagation speed within the resonance frequency range. Simulations were conducted in 5-layer FFNs. Propagation speed measured in cycles per layer as a function of the inter-pulse interval. Bottom/center/top box lines: first/second/third quartiles of the speed distributions. Top/bottom whiskers: largest/smallest value within 1.5 IQR of the upper/lower quartile. Crosses: values observed outside the 1.5 IQR.

This would confirm that CTR is by definition a slow mode of communication and therefore it is not suited for the communication of signals which have to propagate across multiple areas within a short period of time. Note that, e.g., in the FFN shown in **Figure 4a**, synchronous activity reached the fifth layer only after approximately 10 stimulation cycles (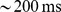 at 40 Hz). We further quantified this result by testing the number of cycles required in a given layer until a significant synchronization level was found in the subsequent layer. A significant degree of synchrony was reached when the instantaneous rate of 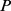 neurons, computed using 5 ms time bins, hit a threshold value equal to 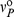 plus five times its s.d.. The results, computed using 100 trials, are shown in **Figure 7** as a function of stimulus frequency (represented as the inter-pulse interval). Our results showed that when stimulated within the main resonance frequency range (39–42 ms intervals) the average speed of propagation was approximately two cycles/layer with small variability. Small deviations from that resonance frequency range resulted in higher trial-to-trial variability of the propagation speed and increased mean while larger deviations resulted in propagation failure.

The results obtained with our example FFN are indicative of how much time it will take to encode a stimulus using CTR at each stage of a processing chain. Naturally, the amount of time will be proportional to the number stages that the activity has to traverse. However, synchrony-based coding using FFNs seems to be suited only for communicating binary signals, i.e., the asynchronous/synchronous activity of a given layer indicates the absence/presence of a particular stimulus (e.g., a specific orientation of a bar of light). By contrast, the encoding of graded signals would require a monotonic relationship between the input and the output of the FFN. We tested the capacity of a diluted FFN to communicate continuous signals using CTR. To this end, we applied periodic stimuli with different amplitude 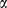 and computed the amplitude response of the network. Our results showed that for these network parameters it was possible to find an input range within which the system's response changed monotonically. Moreover the response remained linear for a restricted range of inputs strength 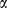 (cf. gray lines in **Figure S2e**). Such a linear operating regime including even a modest degree of saturation, could allow for the communication of graded signals.

We note that our model supports communication of activity between areas that have similar resonance profiles. This automatically ensures selective communication and gives possibility of gating the propagation by small change in the resonance frequency of a network. The experiments proposed above could demonstrate, whether CTR is in principle compatible with the neuronal hardware and physiology, even though they will not necessarily rule out other proposed mechanisms like CTC [17].

## Discussion

Here we propose a novel mechanism for propagation of synchronous spiking activity within weakly coupled FFNs based on the presence of resonance in 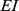 networks. In our model, resonance is a network property that emerges due to the interactions between excitatory and inhibitory neurons in each FFN layer. Using numerical simulations of spiking neuronal networks, we show that a weak and sustained stimulus can be gradually amplified in every layer, thereby overcoming the limitations of synchrony transmission imposed by the diluted interlayer connectivity. We refer to this mode of synchronous activity propagation as “communication through resonance” (CTR).

Until recently, resonance was considered mostly at the level of single cells in both experimental [45]–[47] and theoretical studies [48], [49]. Now, there is increasing experimental evidence showing that resonance also exists at the network level in inhibitory [43] as well as excitatory neuronal populations [50], and may play a crucial role in the generation of cortical rhythms. Theoretical studies have shown that resonance is a fundamental property of 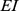 networks [20] and could be used to gate neuronal signals [51].

In our model, such 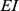 network resonance is used to enable the propagation of synchronous spiking activity in diluted FFNs. In previous theoretical studies, propagation of neuronal activity was restricted to either densely and weakly connected FFNs, which promote the propagation of synchronous activity [7], [9], [42], [52], [53], or sparsely and strongly connected FFNs, which are capable of propagating asynchronous firing ([54], [55]; see [14] for a review). However, biological neuronal networks are typically neither densely connected nor have strong synapses [11] and therefore the mechanisms that govern the propagation of neuronal activity in dense/strong FFNs are not always applicable. Our results indicate that propagation is possible in diluted FFNs, when aided by network resonance, but is restricted to synchronous activity.

Oscillations in the gamma range (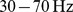), which are a key feature of task-related population activity in several brain areas [56], [57], have emerged as a prominent mechanism that may facilitate propagation of synchronous spiking activity in weakly connected networks [15]. These oscillations can synchronize neuronal activity and provide appropriate temporal windows of excitability, which enable communication between different brain areas. Within these temporal windows, effective functional connections are generated where otherwise only weak structural links may exist [17], [58]. This mode of propagation, however, requires communicating brain areas to oscillate with matched phase and frequency (i.e., their oscillations are coherent) such that synchronous activity from the sender can reach the receiver during its excitable phase and maximize its spiking response. It is commonly believed that coherent oscillations are generated by two independent mechanisms, one responsible for the local generation of oscillations [59] and another mechanism that can flexibly modulate the coherence between spatially distant oscillators [19]. However, the precise nature of the process responsible for achieving such long-range coherence still remains elusive.

Here, we argue that coherent oscillations arise due to the propagation of periodic synchronous spiking activity. In our model, weak rhythmic synchronization provided by the input initially fails to propagate further down the FFN due to the diluted connectivity. The crucial role of the oscillations is to amplify this weak synchronous stimulus by promoting resonance dynamics of the receiving network and enable its propagation across the FFN. This is in contrast to the idea that oscillations are generated independently at every layer and locally synchronize unstructured background input. Our results show that oscillations arise in the network as a consequence of the stimulus propagation, and at the same time the stimulus exploits these oscillations to propagate. Due to this propagation, oscillations in each layer are driven by the previous layer and are hence naturally coherent with a phase that is determined by the conduction delay between the layers [17]. From this perspective coherence becomes a side effect of the propagation dynamics. Thus, a separation of distinct mechanisms that create oscillations and provide coherence is not necessary, as both arise naturally as consequence of CTR. Indeed, recent experimental studies suggest that there is an unidirectional entrainment of coherent oscillations between areas [60]–[62], making the feedforward spread of coherent oscillatory activity, as explained by our model, biologically plausible.

We show that while CTR still works for moderate deviations from periodicity, it is most efficient for propagating periodic stimuli. Notably, the same FFN architecture can transform a sustained firing rate signal into a weak rhythmic stimulus that can then be propagated. Even though it can be argued that environmental stimuli are often not periodic, it has been recently suggested that sensory information could be actively converted into periodic signals by sensing organisms [63], [64].

CTR requires amplification of activity in each layer and, as a consequence, the propagation is slow requiring several cycles to reach the target network. The numbers of cycles needed to transmit synchronous activity across the entire FFN is a function of the connectivity strength between the layers. As the synaptic weights become stronger, the number of cycles required to spread synchrony to the final layer of the FFN decreases and transmission becomes more reliable. Once the weights are sufficiently strong, synchrony flows through the network in one oscillation cycle, which is equivalent to the propagation of synchronous activity in dense/strong connected FFNs investigated by previous studies (cf. [14] for a review). Thus, CTR could generate FFNs with strong connections capable of propagating isolated synchronous events, when certain types of synaptic plasticity are recruited to strengthen the synapses between the different FFN layers. Indeed, coherent oscillations, like those generated by CTR, can provide an ideal dynamical environment to promote synaptic potentiation [65]. In this way, CTR could be regarded as an initial means to propagate activity before strong connections have been formed, while providing the ideal substrate for the generation of fast and reliable communication channels.

In the present study, we describe activity propagation in single FFNs. However, other more complicated network architectures in which multiple FFNs interact may also be possible. In such a scenario, the input could create a stronger response in one such FFN, while partially and weakly activating other FFNs with unmatched resonance frequencies, thereby generating a broadband increase in power around the resonance frequency of the activated FFN. Thus, such a scheme could indeed explain the increase in broadband gamma power of the LFP signal observed during behavioral tasks [66].

Signal gating is an intrinsic property of CTR, since in a given FFN only the stimuli that match its resonance frequency are able to propagate. Selective gating of signals through network resonance has been suggested by previous theoretical studies [51], [67]. Interestingly, the resonance frequency of the network can be dynamically modulated offering the possibility to gate signals differently in time. In our study, we show that modifying the level of external excitation shifts the resonance frequency of the FFN. Additionally, other mechanisms such as neuromodulator mediated changes of the effective connectivity within each layer can have similar effects on the resonance properties of the network. Another alternative gating mechanism is the use of gating signals [14]. Gating activity in dense/strong FFNs requires highly precise and strong gating signals [53]. However, the fact that in CTR the initial phase of the propagation in a given layer is characterized by low amplitude synchrony, which is still insufficient to elicit responses in the next layer, makes CTR suited for a gating mechanism that utilizes relatively imprecise and weak gating signals. Thus, overall CTR constitutes a flexible process that could implement complex spatio-temporal routing of neuronal signals.

As we show here, the dynamical properties of the background activity affect the quality (SNR) of the neuronal signals that are communicated using CTR. More specifically, SNR at low frequency stimulation (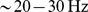) was maximized when background activity state was asynchronous-irregular. This result is in line with experimental evidence which found oscillations in the gamma range to be associated with cortical desynchronization [68], [69]. In contrast, the propagation of 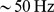 stimuli was successful only when ongoing activity was in a synchronous-irregular state. These findings hint at a hypothetical scenario in which slow periodic modulations of the background dynamics could rhythmically improve or even gate signals that propagate using fast oscillations. The fact that the nesting of slow and fast cortical oscillations (e.g., beta-gamma) is commonly found in experiments (see [70] for a review) could be indicative of such a collaborative effort between different cortical rhythms. These findings open up the possibility that top-down signals may provide the change of background activity state required for coherent feedforward oscillations to be generated.

Importantly, CTR is not restricted to the specific neuron and network model used in this work. The resonance mechanism, which is the essence of the model, is a general property of recurrently connected populations of excitatory and inhibitory neurons [20] and therefore it is widely applicable. Notably, a specific range of propagating frequencies can be achieved by a proper selection of network parameters. In summary, we have shown that communication of neuronal signals across weakly connected networks can be achieved by combining oscillatory activity with resonance dynamics.

## Supporting Information

Figure S1
**Response of isolated layers with different values of** 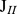 **.** (**a-d**) The reduction of recurrent inhibitory conductance was compensated with an additional external inhibitory Poisson input (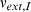) with rate as indicated in the legend. (**a**) Resonance curves for different 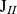 values. Activity is expressed using 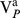 normalized by the mean of the spike count vectors calculated with a time bin of 5 ms (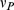). (**b**) Changes in size and frequency of the two main resonance peaks in (a). Blue and red circle indicate first (10–30 Hz) and second (30–80 Hz) main resonance peaks, respectively. (**c**) Pulse triggered average modulation of the inhibitory conductance of 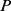 neurons for different 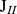 values. (**d**) Pulse triggered average modulation of the membrane potential of 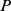 neurons for different 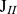 values.(EPS)

Figure S2
**Response of an isolated layer to pulse packets with different** 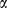 **values**. (**a**) Resonance curves for different 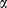 values. Activity is expressed using 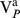 normalized by the mean of the spike count vectors calculated with a time bin of 5 ms (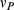). (**b**) Frequency change of the two main resonance peaks in (a). Blue and red circle indicate first (20–30 Hz) and second (50–80 Hz) main resonance peaks, respectively. (**c**) Pulse triggered average modulation of the inhibitory conductance of 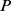 neurons for different 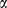 values. (**d**) Pulse triggered average modulation of the membrane potential of 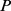 neurons for different 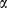 values. (**e**) Amplitude transfer function. Amplitude change of the two main resonance peaks in (a). Colors are same as in (b). Gray lines show a linear approximation to the amplitude change within a range of input values corresponding to 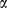 between 15 and 25 spikes.(EPS)

Figure S3
**Alternative FFN architectures that support CTR.** (**a**) All 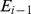 neurons were equally likely to establish long-range connections with the next layer but only 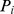 neurons were allowed to receive projections from the previous layer. 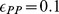 which implied effectively increasing the total number of connections with respect to the architecture used in the main text from 9,000 to 30,000 synapses. The plots bellow the schematic drawing of the FFN are analogous to the ones used in Figure 4b in the main text. (**b**) In contrast to (a), all 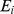 neurons were allowed to receive afferents from the downstream layer and project to the next layer. The total number of connections was kept as in (a) which implied a low 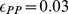.(EPS)

Figure S4
**Effect of longer intra-layer delays and aperiodic input trains on CTR.** (**a**) Effect of longer intra-layer delays on CTR. Delays: 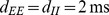 and 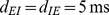. Other parameters as in Figure 4b. Plot structure as in Figure 4b. (**b**) CTR observed during stimulation with strongly jittered pulse packets. Pulse packets: 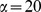 spikes, 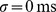 and 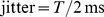, where 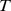 is the interval between pulses. Structure of the panel as in (a).(EPS)

Figure S5
**CTR during synchronous states.** (**a**) Propagation of activity across a 10-layer FFN for different values of 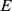 drive. Color code: last layer for which a significant increase in 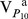 was detected. (**b**) Propagation of synchronous activity in a 10-layer FFN for different stimulus frequencies with 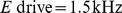. Plot structure as in Figure 4b.(EPS)

Figure S6
**Effect of sinusoidally modulated Poisson inputs in an isolated layer.** (**a**) Layer's spiking and sub-threshold activity as a function of time. Stimulus is a sinusoidally modulated Poisson spike train with parameters: 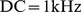; 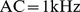; 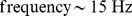. Subpanels as in Figure 3b (**b**) Resonance curves for different AC input values. Activity is measured as in Figure S1.(EPS)

## References

[1] Azouz R, Gray CM (2000) Dynamic spike threshold reveals a mechanism for synaptic coincidence detection in cortical neurons in vivo. Proc Natl Acad Sci U S A 97: 8110–8115. 10859358 10.1073/pnas.130200797 PMC16678

[2] Lger JF, Stern EA, Aertsen A, Heck D (2005) Synaptic integration in rat frontal cortex shaped by network activity. J Neurophysiol 93: 281–293. 15306631 10.1152/jn.00067.2003

[3] Bruno RM, Sakmann B (2006) Cortex is driven by weak but synchronously active thalamocortical synapses. Science 312: 1622–1627. 16778049 10.1126/science.1124593

[4] Rossant C, Leijon S, Magnusson AK, Brette R (2011) Sensitivity of noisy neurons to coincident inputs. J Neurosci 31: 17193–17206. 22114286 10.1523/JNEUROSCI.2482-11.2011 PMC6623863

[5] Abeles M (1982) Role of the cortical neuron: integrator or coincidence detector? Isr J Med Sci 18: 83–92. 6279540

[6] Abeles M (1991) Corticonics: Neural Circuits of the Cerebral Cortex. Cambridge University Press, first edition.

[7] Diesmann M, Gewaltig MO, Aertsen A (1999) Stable propagation of synchronous spiking in cortical neural networks. Nature 402: 529–533. 10591212 10.1038/990101

[8] Reyes AD (2003) Synchrony-dependent propagation of firing rate in iteratively constructed networks in vitro. Nat Neurosci 6: 593–599. 12730700 10.1038/nn1056

[9] Kumar A, Rotter S, Aertsen A (2008) Conditions for propagating synchronous spiking and asynchronous firing rates in a cortical network model. J Neurosci 28: 5268–5280. 18480283 10.1523/JNEUROSCI.2542-07.2008 PMC6670637

[10] Rosenbaum R, Trousdale J, Josic K (2010) Pooling and correlated neural activity. Front Comput Neurosci 4: 9 10.3389/fncom.2010.00009 20485451 PMC2870944

[11] Braitenberg V, Schüz A (1998) Cortex: Statistics and Geometry of Neuronal Connectivity. Heidelberg, Germany: Springer-Verlag, second edition, 249 pp.

[12] Matsumura M, Chen Df, Sawaguchi T, Kubota K, Fetz EE (1996) Synaptic interactions between primate precentral cortex neurons revealed by spike-triggered averaging of intracellular membrane potentials in vivo. J Neurosci 16: 7757–7767. 8922431 10.1523/JNEUROSCI.16-23-07757.1996 PMC6579078

[13] Schrader S, Grüen S, Diesmann M, Gerstein GL (2008) Detecting synfire chain activity using massively parallel spike train recording. J Neurophysiol 100: 2165–2176. 18632888 10.1152/jn.01245.2007 PMC2576207

[14] Kumar A, Rotter S, Aertsen A (2010) Spiking activity propagation in neuronal networks: reconciling different perspectives on neural coding. Nat Rev Neurosci 11: 615–627. 20725095 10.1038/nrn2886

[15] Fries P (2009) Neuronal gamma-band synchronization as a fundamental process in cortical computation. Annu Rev Neurosci 32: 209–224. 19400723 10.1146/annurev.neuro.051508.135603

[16] Engel AK, Fries P, Singer W (2001) Dynamic predictions: oscillations and synchrony in top-down processing. Nat Rev Neurosci 2: 704–716. 11584308 10.1038/35094565

[17] Fries P (2005) A mechanism for cognitive dynamics: neuronal communication through neuronal coherence. Trends Cogn Sci 9: 474–480. 16150631 10.1016/j.tics.2005.08.011

[18] Womelsdorf T, Fries P (2007) The role of neuronal synchronization in selective attention. Curr Opin Neurobiol 17: 154–160. 17306527 10.1016/j.conb.2007.02.002

[19] Uhlhaas PJ, Pipa G, Lima B, Melloni L, Neuenschwander S, et al (2009) Neural synchrony in cortical networks: history, concept and current status. Front Integr Neurosci 3: 17. 19668703 10.3389/neuro.07.017.2009 PMC2723047

[20] Ledoux E, Brunel N (2011) Dynamics of networks of excitatory and inhibitory neurons in response to time-dependent inputs. Front Comput Neurosci 5: 25. 21647353 10.3389/fncom.2011.00025 PMC3103906

[21] Gerstner W, Kistler WM (2002) Spiking Neuron Models: Single Neurons, Populations, Plasticity. 494 pp.

[22] Gewaltig MO, Diesmann M (2007) Nest (neural simulation tool). Scholarpedia 2: 1430.

[23] Eppler JM, Helias M, Muller E, Diesmann M, Gewaltig MO (2008) Pynest: A convenient interface to the nest simulator. Front Neuroinform 2: 12. 19198667 10.3389/neuro.11.012.2008 PMC2636900

[24] Hunter JD (2007) Matplotlib: A 2d graphics environment. Comput Sci Eng 9: 90–95.

[25] van Vreeswijk C, Sompolinsky H (1996) Chaos in neuronal networks with balanced excitatory and inhibitory activity. Science 274: 1724–1726. 8939866 10.1126/science.274.5293.1724

[26] Brunel N (2000) Dynamics of sparsely connected networks of excitatory and inhibitory spiking neurons. J Comput Neurosci 8: 183–208. 10809012 10.1023/a:1008925309027

[27] Softky WR, Koch C (1993) The highly irregular firing of cortical cells is inconsistent with temporal integration of random epsps. J Neurosci 13: 334–350. 8423479 10.1523/JNEUROSCI.13-01-00334.1993 PMC6576320

[28] Barth AL, Poulet JFA (2012) Experimental evidence for sparse firing in the neocortex. Trends Neurosci 35: 345–355. 22579264 10.1016/j.tins.2012.03.008

[29] Ecker AS, Berens P, Keliris GA, Bethge M, Logothetis NK, et al (2010) Decorrelated neuronal firing in cortical microcircuits. Science 327: 584–587. 20110506 10.1126/science.1179867

[30] Harris KD, Thiele A (2011) Cortical state and attention. Nat Rev Neurosci 12: 509–523. 21829219 10.1038/nrn3084 PMC3324821

[31] Borg-Graham LJ, Monier C, Fregnac Y (1998) Visual input evokes transient and strong shunting inhibition in visual cortical neurons. Nature 393: 369–373. 9620800 10.1038/30735

[32] Vida I, Bartos M, Jonas P (2006) Shunting inhibition improves robustness of gamma oscillations in hippocampal interneuron networks by homogenizing firing rates. Neuron 49: 107–117. 16387643 10.1016/j.neuron.2005.11.036

[33] Bartos M, Vida I, Jonas P (2007) Synaptic mechanisms of synchronized gamma oscillations in inhibitory interneuron networks. Nat Rev Neurosci 8: 45–56. 17180162 10.1038/nrn2044

[34] Brunel N, Wang XJ (2003) What determines the frequency of fast network oscillations with irregular neural discharges? i. synaptic dynamics and excitation-inhibition balance. J Neurophysiol 90: 415–430. 12611969 10.1152/jn.01095.2002

[35] Wang XJ (2010) Neurophysiological and computational principles of cortical rhythms in cognition. Physiol Rev 90: 1195–1268. 20664082 10.1152/physrev.00035.2008 PMC2923921

[36] Gray CM, König P, Engel AK, Singer W, et al (1989) Oscillatory responses in cat visual cortex exhibit inter-columnar synchronization which reflects global stimulus properties. Nature 338: 334–337. 2922061 10.1038/338334a0

[37] Burns SP, Xing D, Shapley RM (2011) Is gamma-band activity in the local field potential of v1 cortex a “clock” or filtered noise? J Neurosci 31: 9658–9664. 21715631 10.1523/JNEUROSCI.0660-11.2011 PMC3518456

[38] Xing D, Shen Y, Burns S, Yeh CI, Shapley R, et al (2012) Stochastic generation of gamma-band activity in primary visual cortex of awake and anesthetized monkeys. J Neurosci 32: 13873–13880a. 23035096 10.1523/JNEUROSCI.5644-11.2012 PMC3752128

[39] Nikoli D, Fries P, Singer W (2013) Gamma oscillations: precise temporal coordination without a metronome. Trends Cogn Sci 17: 54–55. 23287106 10.1016/j.tics.2012.12.003

[40] Kumar A, Schrader S, Aertsen A, Rotter S (2008) The high-conductance state of cortical networks. Neural Comput 20: 1–43. 18044999 10.1162/neco.2008.20.1.1

[41] Steriade M, Timofeev I, Grenier F (2001) Natural waking and sleep states: a view from inside neocortical neurons. J Neurophysiol 85: 1969–1985. 11353014 10.1152/jn.2001.85.5.1969

[42] Mehring C, Hehl U, Kubo M, Diesmann M, Aertsen A (2003) Activity dynamics and propagation of synchronous spiking in locally connected random networks. Biol Cybern 88: 395–408. 12750902 10.1007/s00422-002-0384-4

[43] Cardin JA, Carlén M, Meletis K, Knoblich U, Zhang F, et al (2009) Driving fast-spiking cells induces gamma rhythm and controls sensory responses. Nature 459: 663–667. 19396156 10.1038/nature08002 PMC3655711

[44] Uhlhaas P, Singer W (2006) Neural synchrony in brain disorders: relevance for cognitive dysfunctions and pathophysiology. Neuron 52: 155–168. 17015233 10.1016/j.neuron.2006.09.020

[45] Llinás RR (1988) The intrinsic electrophysiological properties of mammalian neurons: insights into central nervous system function. Science 242: 1654–1664. 3059497 10.1126/science.3059497

[46] Steriade M (2000) Corticothalamic resonance, states of vigilance and mentation. Neuroscience 101: 243–276. 11074149 10.1016/s0306-4522(00)00353-5

[47] Dwyer J, Lee H, Martell A, van Drongelen W (2012) Resonance in neocortical neurons and networks. Eur J Neurosci 36: 3698–3708. 23009328 10.1111/ejn.12001

[48] Izhikevich EM (2001) Resonate-and-fire neurons. Neural Netw 14: 883–894. 11665779 10.1016/s0893-6080(01)00078-8

[49] Moca VV, Nikolić D, Singer W, Mureşan RC (2014) Membrane resonance enables stable and robust gamma oscillations. Cerebral Cortex 24: 119–142. 23042733 10.1093/cercor/bhs293 PMC3862267

[50] Lepousez G, Lledo PM (2013) Odor discrimination requires proper olfactory fast oscillations in awake mice. Neuron 80: 1010–1024. 24139818 10.1016/j.neuron.2013.07.025

[51] Akam T, Kullmann DM (2010) Oscillations and filtering networks support flexible routing of information. Neuron 67: 308–320. 20670837 10.1016/j.neuron.2010.06.019 PMC3125699

[52] Gewaltig MO, Diesmann M, Aertsen A (2001) Propagation of cortical synfire activity: survival probability in single trials and stability in the mean. Neural Netw 14: 657–673. 11665761 10.1016/s0893-6080(01)00070-3

[53] Kremkow J, Aertsen A, Kumar A (2010) Gating of signal propagation in spiking neural networks by balanced and correlated excitation and inhibition. J Neurosci 30: 15760–15768. 21106815 10.1523/JNEUROSCI.3874-10.2010 PMC6633769

[54] van Rossum MCW, Turrigiano GG, Nelson SB (2002) Fast propagation of firing rates through layered networks of noisy neurons. J Neurosci 22: 1956–1966. 11880526 10.1523/JNEUROSCI.22-05-01956.2002 PMC6758872

[55] Vogels TP, Abbott LF (2005) Signal propagation and logic gating in networks of integrate-and-fire neurons. J Neurosci 25: 10786–10795. 16291952 10.1523/JNEUROSCI.3508-05.2005 PMC6725859

[56] Varela F, Lachaux JP, Rodriguez E, Martinerie J (2001) The brainweb: phase synchronization and large-scale integration. Nat Rev Neurosci 2: 229–239. 11283746 10.1038/35067550

[57] Tallon-Baudry C (2009) The roles of gamma-band oscillatory synchrony in human visual cognition. Front Biosci 14: 321–332. 10.2741/3246 19273069

[58] Vinck M, Womelsdorf T, Fries P (2013) Gamma-band synchronization and information transmission. In: Principles of Neural Coding, CRC Press Taylor & Francis. pp. 449–469.

[59] Buzsáki G, Wang XJ (2012) Mechanisms of gamma oscillations. Annu Rev Neurosci 35: 203–225. 22443509 10.1146/annurev-neuro-062111-150444 PMC4049541

[60] Gregoriou GG, Gotts SJ, Zhou H, Desimone R (2009) High-frequency, long-range coupling between prefrontal and visual cortex during attention. Science 324: 1207–1210. 19478185 10.1126/science.1171402 PMC2849291

[61] Bosman CA, Schoffelen J, Brunet N, Oostenveld R, Bastos AM, et al (2012) Attentional stimulus selection through selective synchronization between monkey visual areas. Neuron 75: 875–888. 22958827 10.1016/j.neuron.2012.06.037 PMC3457649

[62] Roberts MJ, Lowet E, Brunet NM, Ter Wal M, Tiesinga P, et al (2013) Robust gamma coherence between macaque v1 and v2 by dynamic frequency matching. Neuron 78: 523–536. 23664617 10.1016/j.neuron.2013.03.003

[63] Ahissar E, Arieli A (2012) Seeing via miniature eye movements: A dynamic hypothesis for vision. Front Comput Neurosci 6: 89 10.3389/fncom.2012.00089 23162458 PMC3492788

[64] Landau AN, Fries P (2012) Attention samples stimuli rhythmically. Curr Biol 22: 1000–1004. 22633805 10.1016/j.cub.2012.03.054

[65] Fell J, Axmacher N (2011) The role of phase synchronization in memory processes. Nat Rev Neurosci 12: 105–118. 21248789 10.1038/nrn2979

[66] Waldert S, Pistohl T, Braun C, Ball T, Aertsen A, et al (2009) A review on directional information in neural signals for brain-machine interfaces. J Physiol (Paris) 103: 244–254. 19665554 10.1016/j.jphysparis.2009.08.007

[67] Akam TE, Kullmann DM (2012) Efficient communication through coherence requires oscillations structured to minimize interference between signals. PLoS Comput Biol 8: e1002760 10.1371/journal.pcbi.1002760 23144603 PMC3493486

[68] Munk MH, Roelfsema PR, Knig P, Engel AK, Singer W (1996) Role of reticular activation in the modulation of intracortical synchronization. Science 272: 271–274. 8602512 10.1126/science.272.5259.271

[69] Herculano-Houzel S, Munk MH, Neuenschwander S, Singer W (1999) Precisely synchronized oscillatory firing patterns require electroencephalographic activation. J Neurosci 19: 3992–4010. 10234029 10.1523/JNEUROSCI.19-10-03992.1999 PMC6782718

[70] Cannon J, McCarthy MM, Lee S, Lee J, Börgers C, et al (2013) Neurosystems: brain rhythms and cognitive processing. Eur J Neurosci 39: 705–19 10.1111/ejn.12453 24329933 PMC4916881

